# The occurrence of spring forms in tetraploid Timopheevi wheat is associated with variation in the first intron of the *VRN-A1* gene

**DOI:** 10.1186/s12870-016-0925-y

**Published:** 2016-11-16

**Authors:** Andrey Borisovich Shcherban, Aleksandra Aleksandrovna Schichkina, Elena Artemovna Salina

**Affiliations:** 1 The Federal Research Center “Institute of Cytology and Genetics of Siberian Branch of the Russian Academy of Sciences”, Lavrentiev ave. 10, Novosibirsk, 630090 Russia; 2 Institute of General Genetics of the Russian Academy of Sciences, Gubkina str., 3, Moscow, 119991 Russia

**Keywords:** Allelic variation, Vernalization, *VRN-1* gene, Promoter, First intron, *Triticum*, *Aegilops*

## Abstract

**Background:**

*Triticum araraticum* and *Triticum timopheevii* are tetraploid species of the Timopheevi group. The former includes both winter and spring forms with a predominance of winter forms, whereas *T. timopheevii* is considered a spring species. In order to clarify the origin of the spring growth habit in *T. timopheevii*, allelic variability of the *VRN-1* gene was investigated in a set of accessions of both tetraploid species, together with the diploid species *Ae. speltoides*, presumed donor of the G genome to these tetraploids.

**Results:**

The promoter region of the *VRN-A1* locus in all studied tetraploid accessions of both *T. araraticum* and *T. timopheevii* represents the previously described allele *VRN-A1f* with a 50 bp deletion near the start codon. Three additional alleles were identified namely, *VRN-A1f-del*, *VRN-A1f-ins* and *VRN-A1f-del/ins,* which contained large mutations in the first (1^st^) intron of *VRN-A1.* The first allele, carrying a deletion of 2.7 kb in a central part of intron 1, occurred in a few accessions of *T. araraticum* and no accessions of *T. timopheevii.* The *VRN-A1f-ins* allele, containing the insertion of a 0.4 kb MITE element about 0.4 kb upstream from the start of intron 1, and allele *VRN-A1f-del/ins* having this insertion coupled with a deletion of 2.7 kb are characteristic only for *T. timopheevii.* Allelic variation at the *VRN-G1* locus includes the previously described allele *VRN-G1a* (with the insertion of a 0.2 kb MITE in the promoter) found in a few accessions of both tetraploid species. We showed that alleles *VRN-A1f-del* and *VRN-G1a* have no association with the spring growth habit, while in all accessions of *T. timopheevii* this habit was associated with the dominant *VRN-A1f-ins* and *VRN-A1f-del/ins* alleles. None of the *Ae. speltoides* accessions included in this study had changes in the promoter or 1^st^ intron regions of *VRN-1* which might confer a spring growth habit. The *VRN-1* promoter sequences analyzed herein and downloaded from databases have been used to construct a phylogram to assess the time of divergence of *Ae. speltoides* in relation to other wheat species.

**Conclusions:**

Among accessions of *T. araraticum*, the preferentially winter predecessor of *T. timopheevii*, two large mutations were found in both *VRN-A1* and *VRN-G1* loci (*VRN-A1f-del* and *VRN-G1a*) that were found to have no effect on vernalization requirements. Spring tetraploid *T. timopheevii* had one *VRN-1* allele in common for two species (*VRN-G1a*), and two that were specific (*VRN-A1f-ins*, *VRN-A1f-del/ins*). The latter alleles include mutations in the 1^st^ intron of *VRN-A1* and also share a 0.4 kb MITE insertion near the start of intron 1. We suggested that this insertion resulted in a spring growth habit in a progenitor of *T. timopheevii* which has probably been selected during subsequent domestication. The phylogram constructed on the basis of the *VRN-1* promoter sequences confirmed the early divergence (~3.5 MYA) of the ancestor(s) of the B/G genomes from *Ae. speltoides*.

**Electronic supplementary material:**

The online version of this article (doi:10.1186/s12870-016-0925-y) contains supplementary material, which is available to authorized users.

## Background

Bread or common wheat, *Triticum aestivum* L., is one of the most important crops, providing a staple food for almost half of the human population world-wide. It is an allohexaploid (BBAADD genome, 2n = 42) that arose through hybridization of tetraploid wheat species (BBAA) with the diploid donor of D- genome. This event occurred about 8000 years ago [1, 2]. Among tetraploid predecessors of common wheat are the emmer wheat *T. dicoccoides* (BBAA, 2n = 28), a wild progenitor from which modern tetraploid and hexaploid cultivated wheats were derived, and a separate group of species which belong to the section Timopheevi A. Filat. et Dorof. (GGAA).

Tetraploid Timopheevi wheats include the closely related species *T. araraticum* Jakubz. and *T. timopheevii* (Zhuk) Zhuk. *T. araraticum* grows primarily in Armenia, Azerbaijan, Georgia, Iran, Iraq and Turkey [3, 4] and originated from natural hybridization between *T. urartu* (AA, 2n = 14) and *Ae. speltoides* (SS, 2n = 14), the proposed donors of A- and G- genomes, respectively [1, 5–7]. *T. araraticum* is the ancestor of the domestic *T. timopheevii.* The latter has a restricted area of origin near Zanduri village in western Georgia [8]. Due to this feature, *T. timopheevii* is characterized not only by morphological homogeneity but also by a low cytogenetic variability [9].

The vernalization requirement, or necessity of cold treatment for the induction of flowering, is one of the most important traits affecting yield and adaptability of wheat crops. This characteristic has been intensively investigated in common wheat *T. aestivum* in which it is mainly controlled by three homoeologous *VRN-1* loci: *VRN-A1*, *VRN-B1* and *VRN-D1* [10–12]. Winter varieties are homozygous for the recessive alleles at the three *VRN-1* loci, whereas spring wheat has dominant alleles at one or more of these loci. The dominant *VRN-1* alleles are usually associated with mutations in two main regulatory regions of the *VRN-1* gene, the promoter and first (1^st^) intron. Such mutations, namely, promoter deletions and insertions, and large deletions in intron 1, were initially detected in diploid *T. monococcum* (AA, 2n = 14), common wheat *T. aestivum* and tetraploid *T. durum* [10, 13, 14].

Recently, we characterized *VRN-1* allelic diversity in a wide set of accessions representing wild tetraploid *T. dicoccoides* and three diploid A genome progenitor species [15]. The data showed that a set of dominant *VRN-1* alleles characteristic of wheat polyploids probably arose after allopolyploidization and were selected during domestication. Tetraploid Timopheevi wheats have not been systematically examined in this respect. One of the most conspicuous differences between *T. araraticum* and *T. timopheevii* is that the former includes both winter and spring forms with predominance of winter forms, whereas *T. timopheevii* is considered a spring species [4, 16]. Hence, *T. timopheevii* represents a good model to study the origin of the spring growth habit in early wheat polyploids, a habit which may be primarily attributed to variation in the *VRN-1* gene.

In order to identify and characterize which alleles of *VRN-1* may affect the vernalization requirement in *T. timopheevii*, in this study we performed a comparative molecular analysis of the main regulatory regions of *VRN-1* in Timopheevi wheat species. We also studied variability within *VRN-1* in the diploid *Ae. speltoides*, the proposed donor of B/G- genome.

## Results

### *VRN-1* allelic variability in tetraploid Timopheevi wheat

#### Promoter region

**Fig. 1:**
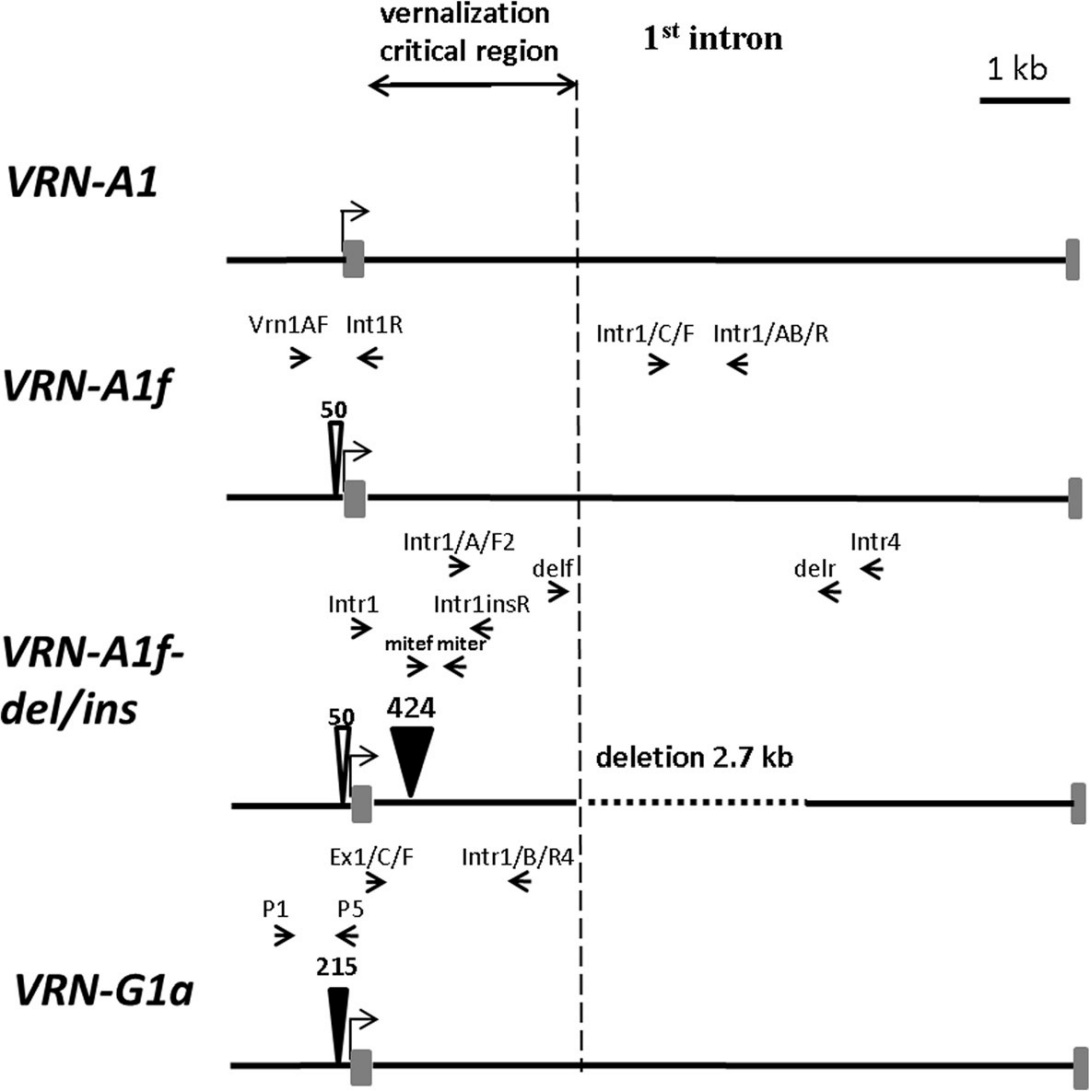
Schematic representation of different *VRN-1* alleles including promoter, 1^st^ exon (grey rectangle) and 1^st^ intron. The positions of specific primers are shown above each scheme. Deletions and insertions of MITE transposable elements are indicated by empty and filled triangles, respectively, with sizes (bp) above

**Fig. 2:**
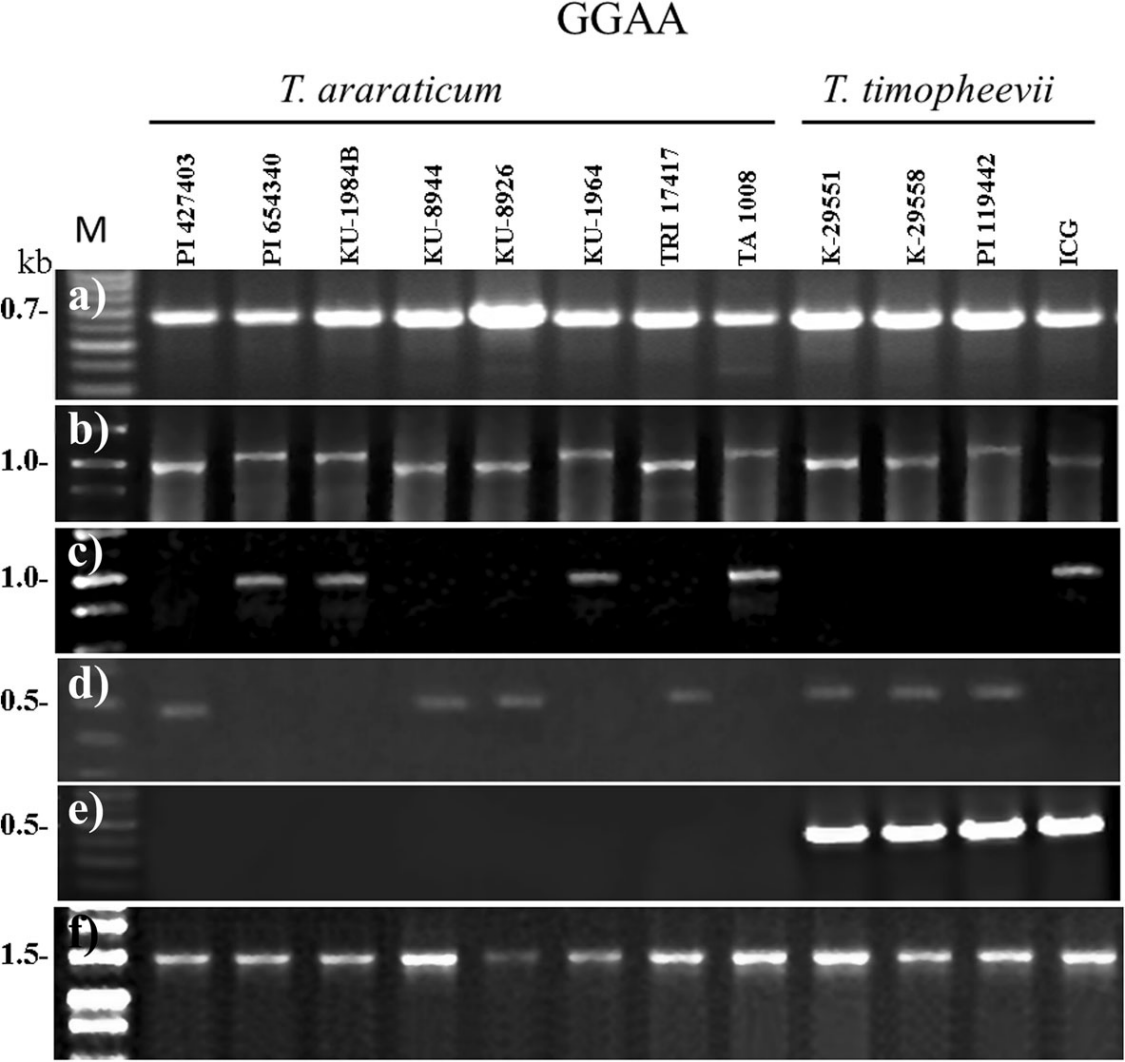
PCR amplification with primers VrnA1F/Int1R (**a**), P1/P5 (**b**), Intr1/C/F // Intr1/AB/R (**c**), delf/delr (**d**), mitef/miter (**e**), Ex1/C/F // Intr1/B/R4 (**f**). Accession numbers, species and genotype are given at the top

Vernalization sensitivity in tetraploid wheat *T. araraticum* and *T. timopheevii* is controlled by alleles at the two homoeologous loci, *VRN-A1* and *VRN-G1*. The specific primers Vrn1AF and Int1R were used to identify variation in the length of the promoter region of the *VRN-A1* locus, as described by Yan et al. [13] (Table 1; Fig. 1). All studied accessions of both tetraploid species yielded a PCR product of approximately 0.65 kb (Additional file 1; Fig. 2a). We sequenced the PCR products obtained with VrnA1F/Int1R primers for a selected set of accessions representing these species (Table 2).

**Table 1:** PCR markers for determining the presence of different alleles of *VRN-A1*, *VRN-G1* in tetraploid Timopheevi wheats and diploid *Ae. speltoides*

PCR marker	Name	Primer (5’ → 3’)	Target allele(s)	Expected pro-duct size (bp)	Annealing temp. (°C)	Reference.
*VRN-A1 *marker^a^	Vrn1AF	GAAAGGAAAAATTCTGCTCG	*VRN-A1f*	655	55	[13]
	Int1R	GCAGGAAATCGAAATCGAAG	*VRN-A1f-del*			
			*VRN-A1f-ins*			
			*VRN-A1f-del/ins*			
*VRN-A1 *Non-deletion	Intr1/C/F	GCACTCCTAACCCACTAACC	*VRN-A1f*	1068	56	[14]
	Intr1/AB/R	TCATCCATCATCAAGGCAAA	*VRN-A1f-ins*			
*VRN-A1 *insertion	Intr 1	ATCATCTTCTCCACCAAGGG	*VRN-A1f*	1480	50	[15]
	Intr1insR	AATGAACAGCACGGAAACAG	*VRN-A1f-ins*	1910		
			*VRN-A1f-del/ins*			
	mitef	CAGGTAAGGTATGAGGTGAC	*VRN-A1f-ins*	370	52	-
	miter	GATTCCAAATGAGAAGATGAGG	*VRN-A1f-del/ins*			
*VRN-A1* deletion of 2.7 kb	Intr1/A/F2	AGCCTCCACGGTTTGAAAGTAA	*VRN-A1f-del*	~2500	56	[14]
	Intr4	GCGCCATTAGGGAGGCACTT	*VRN-A1f-del/ins*			
	delf	ACATGTAAGCAGATCCTATCGA	*VRN-A1f-del*	450	53	-
	delr	TGCTTTAGATCTTTCTTCACGG	*VRN-A1f-del/ins*			
*VRN-G1* marker^a^						
for *T. araraticum*/	P1	TACCCCTGCTACCAGTGCCT	*VRN-G1*	900	55	[23]
*T. timopheevii*	P5	GGCCAACCCTACACCCCAAG	*VRN-G1a*	1100		
for *Ae. speltoides*	P8	CTAGGACTGGCGAGTATCTT	*VRN-1*	900	55	-
	P10	GAGAACCGGGCCAACCCTAC				
*VRN-G1* Non-deletion						
for *T. araraticum*/	Ex1/C/F	GTTCTCCACCGAGTCATGGT	*VRN-G1*	1530	56	[14]
*T. timopheevii*	Intr1/B/R4	CAAATGAAAAGGAATGAGAGCA	*VRN-G1a*			
for *Ae. speltoides*	Ex1/C/F	GTTCTCCACCGAGTCATGGT	*VRN-1*	1530	60	[14]
	Intr1/D/R4	AAATGAAAAGGAACGAGAGCG				

^a^These diagnostic markers detect allelic variation at the promoter regions. In other cases variation within intron 1 of corresponding genes is detected

**Table 2:** Allelic variants of *VRN-A1* and *VRN-G1* genes in a selected set of accessions of tetraploid Timopheevi wheats

Species (genome)	Accession number (origin)	Growth type (Winter/ Spring)	*VRN-A1* ^a^			*VRN-A1* alleles	*VRN-G1* ^a^		*VRN-G1* alleles	GenBank Ac.№
			Promoter	Intron 1			Promoter	Intron 1 (*vrn-G1*)		
				Insertion 0.4 kb	Deletion 2.7 kb					
							Insertion 0.2 kb			
*T. araraticum* (GGAA)	PI 427403 (Iraq)	W	*VRN-A1f*	*-*	*+*	*VRN-A1f-del*	*-*	+	*VRN-G1*	*VRN-G1*: KX344116
	PI 654340 (Turkey)	W	*VRN-A1f*	*-*	*-*	*VRN-A1f*	*+*	+	*VRN-G1a*	*VRN-A1f*: KX344106; *VRN-G1a*: KX344117
	KU-1984B (Turkey)	W	*VRN-A1f*	-	-	*VRN-A1f*	+	+	*VRN-G1a*	*VRN-A1f*: KX344101
	KU-8944 (Iran)	W	*VRN-A1f*	*-*	*+*	*VRN-A1f-del*	-	+	*VRN-G1*	-
	KU-8926 (Turkey)	W	*VRN-A1f*	-	+	*VRN-A1f-del*	-	+	*VRN-G1*	-
	KU-1964 (Turkey)	W	*VRN-A1f*	*-*	*-*	*VRN-A1f*	+	+	*VRN-G1a*	*VRN-A1f*: KX344102
	TRI 17417 (Azer.)	W	*VRN-A1f*	*-*	*+*	*VRN-A1f-del*	-	+	*VRN-G1*	-
	TA 1008 (Turkey)	W	*VRN-A1f*	*-*	*-*	*VRN-A1f*	+	+	*VRN-G1a*	*VRN-A1f*: KX344103
*T. timopheevii* (GGAA)	K-29551 (Georgia)	S	*VRN-A1f*	*+*	+	*VRN-A1f-del/ins*	-	+	*VRN-G1*	-
	K-29558 (Georgia)	S	*VRN-A1f*	*+*	*+*	*VRN-A1f-del/ins*	-	+	*VRN-G1*	-
	PI 119442 (Turkey)	S	*VRN-A1f*	+	+	*VRN-A1f-del/ins*	+	+	*VRN-G1a*	*VRN-A1f*: KX344107; *VRN-G1a*: KX344118
	ICG (unknown)	S	*VRN-A1f*	*+*	*-*	*VRN-A1f-ins*	-	+	*VRN-G1*	*VRN-A1f*: KX344108

^a^In all cases the promoter region was sequenced; insertion and deletions in intron 1 were determined by PCR (see Table 1) and partial sequencing. In the case of *VRN-G1* the structure of intron 1 corresponds to the recessive form of the gene

The promoter sequences of all studied accessions were 99 % homologous to each other and to the previously published *VRN-A1f* promoters from *T. timopheevii* and *T. araraticum* (GQ451751-451753 and GQ451762-451765, respectively). Compared to the other known *VRN-A1* sequences, the *VRN-A1f* promoter has a 50 bp deletion in the position -63 bp from the start codon (Fig. 1). A few indels were also identified between the studied sequences, including a 1 bp deletion (C nucleotide within C-rich segment upstream from the CArG-box) in 4 out of 8 accessions of *T. araraticum* and four nucleotide substitutions*.* The four studied *VRN-A1* promoter sequences of *T. timopheevii* have no specific indels compared to the respective sequences of *T. araraticum.*

**Fig. 3:**
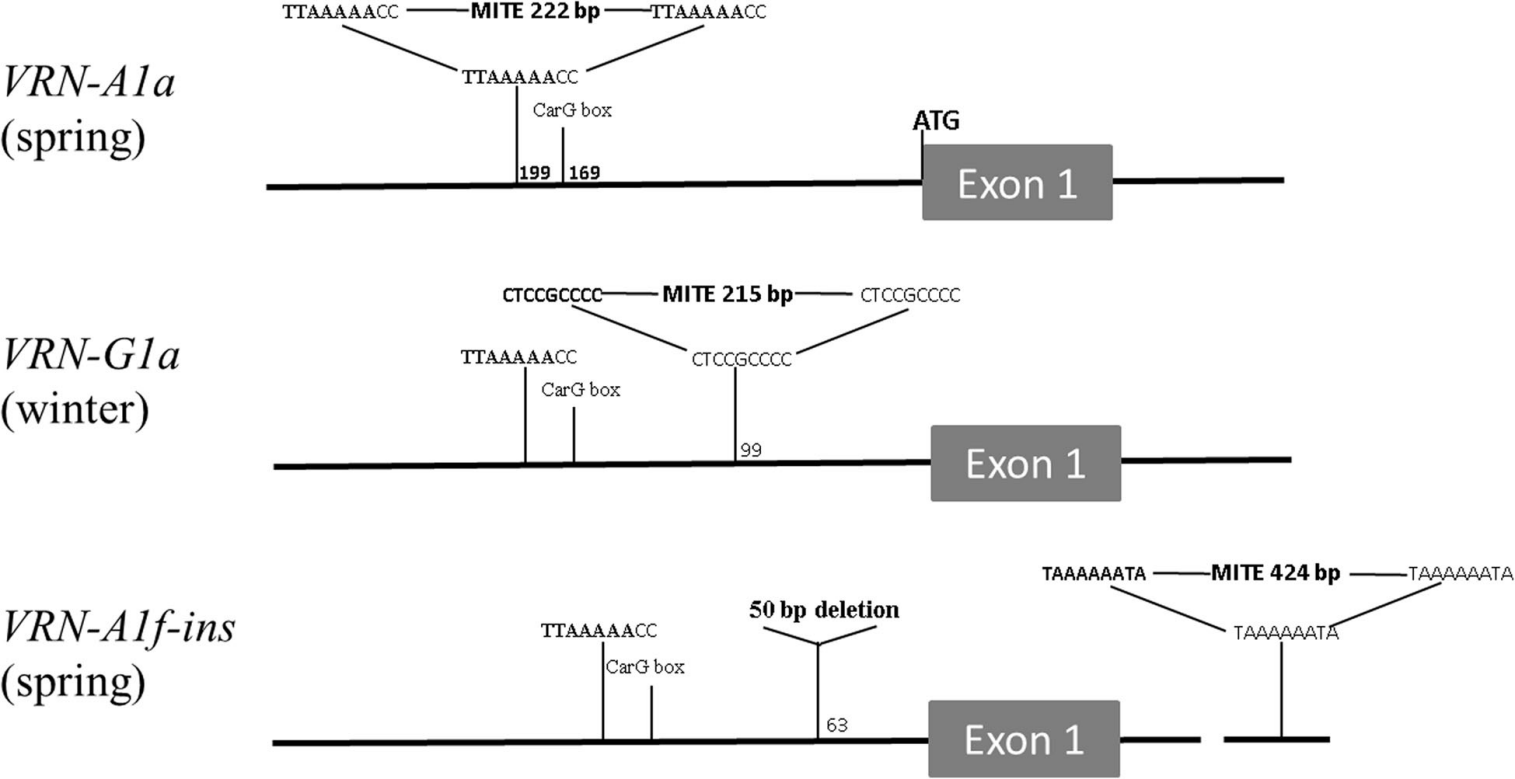
Schemes of different *VRN-1* alleles with insertions of MITE elements indicated. The putative TATA- box (TTAAAAA) and CArG-box are depicted. The exact position of indels and regulatory sites within the promoter region is marked by the number of bases counted from the start codon

For analysis of the *VRN-G1* locus, the primers P1/P5 were used to amplify an approximately 0.9 kb region of the promoter sequence (Table 1; Fig. 1). 41 accessions of *T. araraticum* and 3 accessions of *T. timopheevii* gave the expected PCR product, while 4 accessions of *T. araraticum* and 1 accession of *T. timopheevii* generated a larger PCR product of about 1.1 kb (Fig. 2b). Sequencing of the product from one of the former accessions (PI 427403) showed that it is highly homologous with the *VRN-G1* promoter from *T. araraticum* (KR055682), while those from accessions PI 654340 and PI 119442,- representatives of the latter group, are almost identical with the previously published *VRN-G1a* promoter from *T. timopheevii* (GQ451755) (Table 2). The last promoter contains a MITE (miniature inverted-repeat transposable element)-like insertion located 99 bp upstream from the stop codon (Fig. 1). The MITE consisted of 215 bp, including a 9 bp host duplication (CTCCGCCCC) and 29 bp inverted repeats at the ends (Fig. 3). The sequence was found to be 92 % identical to a foldback element inserted in the *VRN-A1a* allele of *T. aestivum* (AY616458). Thus, the recessive *VRN-G1* allele is characteristic of most *T. araraticum* and *T. timopheevii* accessions, whereas the *VRN-G1a* allele occurs with less frequency in both species.

#### 1^st^ intron

The primer pair Intr1/C/F and Intr1/AB/R for the 1^st^ intron of *VRN-A1* (Table 1; Fig. 1) were previously used to confirm the presence of the recessive allele of *T. aestivum* with no significant changes (deletions or insertions) in intron 1 [14]. 41 accessions of *T. araraticum* (including 4 accessions represented in Fig. 2) and one accession of *T. timopheevii* yielded a PCR product of approximately 1 kb, indicating the presence of the intact 1^st^ intron within the *VRN-A1* gene (Fig. 2c). The remaining accessions of both species gave no PCR products with this pair of primers. To identify the possible changes explaining this last pattern, the 1^st^ intron region of *VRN-A1* was split into subregions which were amplified separately by different combinations of primers.

The central portion of intron 1 flanked by the primers Intr1/A/F2 and Intr4 (Table 1; Fig. 1) can hardly be amplified in the case of the recessive *VRN-A1* allele due to its large size (~5 kb). However, for four *T. araraticum* and three *T. timopheevii* accessions a PCR product of approximately 2.5 kb was amplified using these primers (data not presented). This indicated the presence of a deletion preventing amplification with pair Intr1/C/F // Intr1/AB/R (Fig. 1). The one-sided sequencing of the product allowed us to establish the boundaries of the deletion and its size- 2.7 kb by comparison with the known recessive *VRN-A1* alleles. The primers delf and delr were designed bordering the deletion and giving a 450 bp fragment (Table 1; Fig. 2d). Hence, the deletion of 2.7 kb in intron 1 of *VRN-A1* is characteristic of three studied accessions of *T. timopheevii* and 4 out of 45 accessions of *T. araraticum* (Table 2).

Using the primer combination Intr1// Intr1insR, all *T. timopheevii* accessions yielded a larger PCR product compared with accessions of *T. araraticum* (data not shown). The sequencing of this product showed the presence of an insertion located 435 bp downstream from the end of exon 1 (Fig. 3). This insertion of 424 bp long was found to be 99 % identical to the previously identified MITE element in the *VRN-A1f-like* allele of *T. militinae* [17]. In order to confirm this result, we used the primer pair mitef/ miter targeting the right junction of the insertion (Table 1; Fig. 1). All accessions of *T. timopheevii* yielded a product of 370 bp, whereas no product was obtained with the *T. araraticum* accessions (Fig. 2e). Therefore, unlike the 2.7 kb deletion, the 0.4 kb MITE insertion in intron 1 is a specific marker for the *VRN-A1* allele of *T. timopheevii.*

Here, we designated the *VRN-A1f* alleles with different mutations in the 1^st^ intron as *VRN-A1f-del*, *VRN-A1f-ins* and *VRN-A1f-del/ins* (the 2.7 kb deletion, the 0.4 kb insertion and both mutations, respectively) (Table 2).

The primer pair Ex1/C/F and Intr1/B/R4 was designed as a positive control for the absence of large mutations in intron 1 of the *VRN-B1/G1* locus [14] (Table 1). Using these primers, all accessions of *T. araraticum* and *T. timopheevii* yielded a 1530 bp PCR product indicating an intact 1^st^ intron region (Fig. 2f).

Thus, the tetraploid wheats *T. araraticum* and *T. timopheevii* displayed most structural variation at the 1^st^ intron region of *VRN-A1*, including the 0.4 kb MITE insertion specific for *T. timopheevii*. Unlike *VRN-A1*, the *VRN-G1* locus showed no variation in the 1^st^ intron, while in the promoter a restricted variation was revealed (allele *VRN-G1a*) in some accessions of both species.

### Evaluation of growth habit in tetraploid accessions

To assess the impact of *VRN-1* alleles on vernalization requirements in tetraploid wheat, we selected the accessions of *T. araraticum* and *T. timopheevii* presented in Table 2. Notably, in all the studied accessions of *T. araraticum* the alleles *VRN-A1f*, *VRN-A1f-del* and *VRN-G1a* were associated with a winter growth habit, implying that these alleles have no effect on vernalization requirements. All four accessions of *T. timopheevii* with alleles *VRN-A1f-del/ins* (insertion + deletion in intron 1) and *VRN-A1f-ins* (insertion in intron 1, no deletion) were spring types. Consequently, the insertion of the MITE element in the 1^st^ intron of *VRN-A1* locus is probably associated with the spring growth habit of *T. timopheevii.*

### *VRN-1* allelic variation in *Ae. speltoides*

#### Promoter region

The primer pair P1 and P5 successfully used for amplification of the *VRN-G1* promoter in *T. araraticum* and *T. timopheevii* (see above) gave no PCR products in the case of *Ae. speltoides.* Primers pair P8/P10 was designed, so that primer P8 anneals close to the 3’-end of the annealing site for P1 primer, while P10 overlaps the 3’-half of the annealing site for P5 (Table 1). This pair yielded an expected product of approximately 0.9 kb in all the studied accessions of *Ae. speltoides* (data not presented)*.* This indicates the presence of the intact *VRN-G1* promoter, except under circumstances where flanking sequences may have been modified, preventing amplification with P1/P5. To further analyze allelic variation in the *VRN-G1* promoter, we randomly selected 7 accessions of *Ae. speltoides* and sequenced the corresponding PCR products obtained with P8/P10 primers.

All 7 sequences were highly similar to each other (≥98 % homology). Minor variation between individual sequences was found, including nucleotide substitutions and small deletions of up to 4 bp. Most of this variation was upstream from the conserved region encompassing about 0.3 kb from the start codon and containing the putative regulatory sites [10, 13, 18]. A conspicuous polymorphism was found in the tandem track of AGCC repeats ~ 0.6 kb upstream from the start codon. All known *VRN-B1/G1* sequences of wheat polyploids contain 4 repeats, while in the studied sequences of *Ae. speltoides* the number of repeats varies from two (K-2278, I-551352, I-570060) up to three (K-453, K-3257, K-911, K-443).

The *VRN-G1* promoter sequences of *Ae. speltoides* displayed a similar homology (92-93 %) to the corresponding sequences of wild tetraploid species *T. araraticum* and *T. dicoccoides* accessed from databases. Sequence variation as compared with tetraploids included a few small deletions and insertions up to 7 bp, the largest deletion of 21 bp located about 0.5 kb upstream from the start codon is characteristic of all studied *Ae. speltoides* sequences.

#### 1^st^ intron

All *Ae. speltoides* accessions gave no the expected PCR product of 1.5 kb using primers Ex1/C/F and Intr1/B/R4 which anneal with exon 1 and intron 1 sequences, respectively. Taking into account the relationship between *Ae. speltoides* and *Ae. tauschii* (DD genome), instead of Intr1/B/R4 we tested the Intr1/D/R4 primer pair having 2 mismatches specific for *Ae. tauschii* in the same annealing site (Table 1). The presence of the expected PCR product in the latter case (data not shown) indicates a divergence within the Intr1/B/R4 annealing site as the cause of the negative result using the first primer pair.

### Phylogenetic analysis of *VRN-1* promoter sequences

**Fig. 4:**
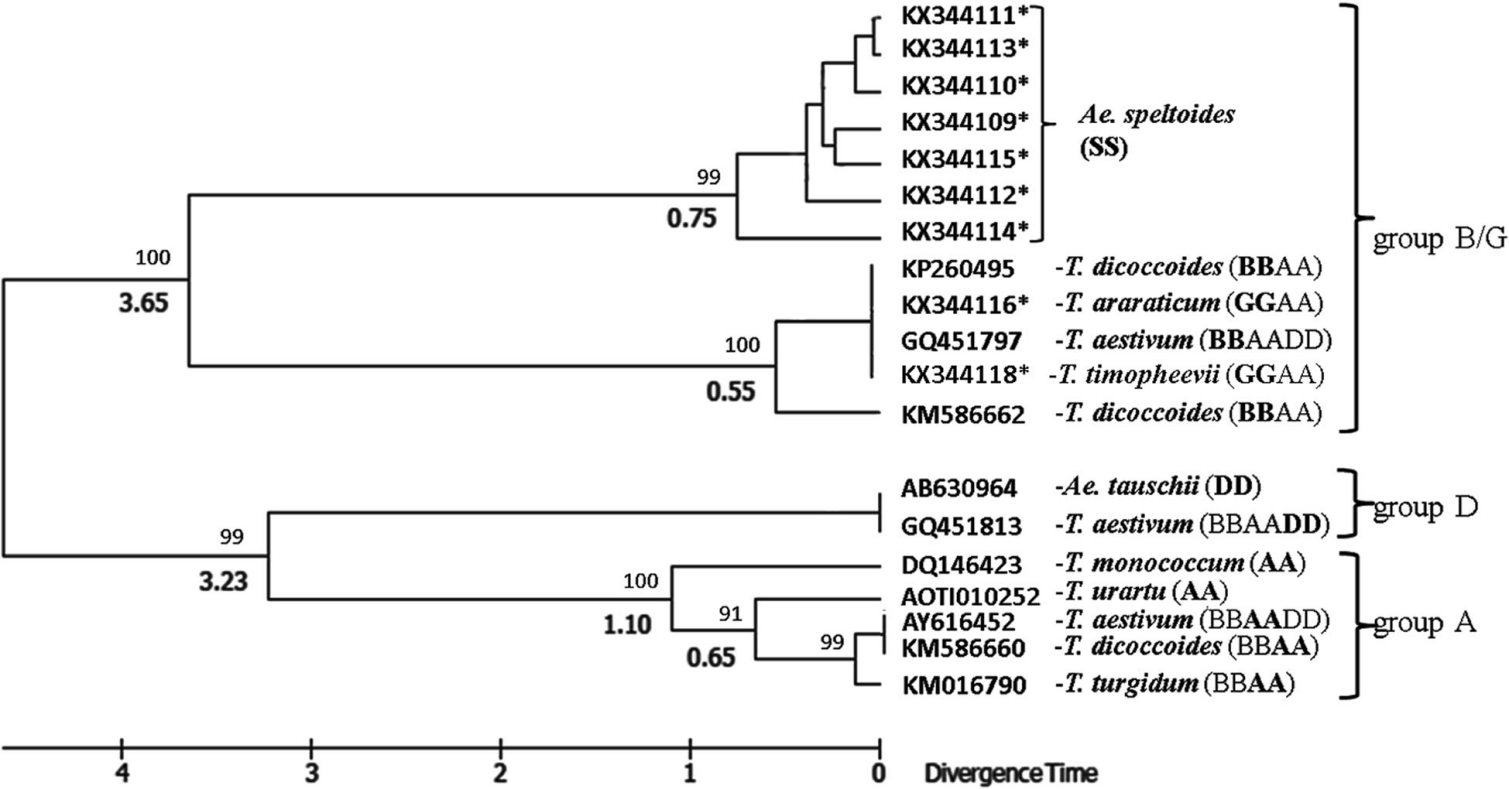
The neighbor-joining phylogram of species based on the alignment of the nucleotide *VRN-1* promoter sequences. Letters in bold within genotypes indicate genomes from which the corresponding sequences were isolated. The numbers above and below forks indicate bootstrap values and times of divergence, respectively. Scale of divergence time in MYA is shown below the phylogram. Sequences were selected covering, at least, 0.8 kb of the promoter region. The sequences analysed herein are marked with an asterisk

To assess the degree of divergence in *VRN-1* promoter sequences between the diploid species that donated their genomes to polyploids, and the different polyploid wheats (tetra-, or hexaploid-), we constructed a neighbor-joining phylogram based on the alignment of the nucleotide sequences obtained in this research and downloaded from databases (see Methods). In general, three main groups form the distinct branches on the tree (Fig. 4). The sequences of *Ae. speltoides* originated from the same branch as those of the B/G- genomes of polyploids, but diverged comparatively early ~ about 3.6 MYA according to our estimation. This date is comparable with the date of divergence of the D-genome group (3.2 MYA) which occupies an intermediate position between B/G and A- groups. The latter falls into a separate branch of the diploid *T. monococcum* and subgroup comprising the diploid *T. urartu* and A-genome sequences of polyploids. Within this subgroup the previously established date of divergence of the allopolyploid branch (0.65 MYA) was used for calibration (see Methods).

## Discussion

High plasticity and adaptability of polyploid wheat to growth at various latitudes is typically provided by allelic variation of genes which control sensitivity to vernalization and photoperiod (*VRN* and *PPD*, respectively). Following extensive molecular genetic studies of cereal plants, the integrated model of flowering regulation including both factors was proposed [19]. According to this model, the *VRN-1* gene plays a key role in combining different signal transduction pathways via two main regulatory regions in its structure, promoter and 1^st^ intron. Mutations in these regions have an impact on *VRN-1* expression and enables plants to significantly modulate vernalization requirements and flowering time [20–24]. However, the specific mechanisms by which each region exerts their effects are still to be established.

### The origin of the spring growth habit in tetraploid Timopheevi wheat

Most of the wild Triticeae species, both diploid and polyploid, have a winter growth habit, suggesting that the recessive *VRN-1* allele is the ancestral form. By contrast, there are many cultivated polyploid wheat varieties with a spring growth habit and with at least one dominant *VRN-1* allele [25]. Polyploid wheat is divided into two evolutionary lineages, Emmer (BBAA) and Timopheevi (GGAA), which formed through hybridization between *T. urartu* and *Ae. spetoides*, and divergence of the first wild tetraploids *T. diccocoides* (BBAA) and *T. araraticum* (GGAA) occurred over the course of several tens of thousands years [26]. The appearance of spring forms in the Emmer group is presumably associated with mutations in the promoter or 1^st^ intron of *VRN-A1* locus, mainly, deletions of different lengths [15]. In order to study the origin of the spring growth habit in Timopheevi wheat, we compared the wild species *T. araraticum* to its domesticated spring descendant, *T. timopheevii*.

The promoter region of *VRN-A1* in both tetraploid species displays a high level of homogeneity and is almost identical to the previously described *VRN-A1f* allele of *T. timopheevii* [27]. The characteristic of this allele is a 50 bp deletion near the start codon (Fig. 1). Such a deletion has not been described in Emmer or diploid wheat species, so it is probably specific for Timopheevi wheat. The *VRN-G1* locus both in the promoter and its 1^st^ intron showed no significant variation between the two tetraploid species. The single *VRN-G1a* allele found with a 0.2 kb insertion has a low frequency among *T. araraticum* accessions studied here (8 %) and was also found in two accessions of *T. timopheevii*, the previously studied GQ451755 and presented here KX344118 (Table 2). Evaluation of growth habits demonstrated that neither *VRN-A1f* nor *VRN-G1a* are responsible for spring type growth, since all accessions carrying these alleles were winter types. Simultaneously, we found an obvious species-specific polymorphism within the 1^st^ intron region of *VRN-A1.* Alterations in this region include the 0.4 kb MITE insertion and the 2.7 kb deletion located about 0.4 kb and ~3 kb downstream from the start of intron 1, respectively (Fig. 1). Recently, Ivanicova et al. [17] described the *VRN-A1f-like* allele containing both mutations in lines of spring bread wheat with introgression of genetic material from *T. militinae* Zhuk. and Migush., the free-threshing mutant that was selected from a single specimen of *T. timopheevii* [28]. These lines were characterized by a significant difference in flowering time- the effect associated with the *VRN-A1f-like* allele. Here, we have demonstrated for the first time that the MITE insertion and the deletion in the 1^st^ intron of *VRN-A1* are of independent origin and have different effects on the growth habit. The deletion of 2.7 kb obviously appeared before the divergence of *T. timopheevii* from *T. araraticum*, since 4 accessions of the last species contained this single mutation (allele *VRN-A1f-del*). In addition, we found the MITE insertion in no accessions of *T. araraticum* and all the studied accessions of *T. timopheevii* where it was combined with the 2.7 kb deletion (*VRN-A1f-del/ins*) in 3 accessions, or present as a single mutation (*VRN-A1f-ins*) in 1 accession (Table 2). Therefore, we suggested that the spring growth habit arose in an ancestor of the modern *T. timopheevii* due to insertion of MITE within the 1^st^ intron of *VRN-A1* and this mutation has been selected during domestication of this species.

Previous data support the importance of intron 1 of *VRN-1* gene in the vernalization response and control of heading time, and the proposed “vernalization critical” regulatory region has been located encompassing ~ 3 kb from the start of intron 1 (Fig. 1) [11, 14]. A number of mutations (deletions, insertions etc.) in this region have been associated with a spring growth habit in different crops [14, 21, 29, 30]. It was suggested that all these mutations may affect epigenetic chromatin states, resulting in a higher basal level of *VRN-1* expression which is necessary for inducing a spring type of growth [22, 30]. Previously, we described the dominant allele *VRN-B1c* as having large alterations (deletion and duplication) within the 5’-flanking part of intron 1 and found that these mutations resulted in a higher level of *VRN-1* transcription and subsequent reduction in flowering time compared to the allele without such mutations [23, 24]. Another example is the *VRN-A1ins* allele of the diploid *T. monococcum* (AA) with a 0.5 kb insertion in the same region which is associated with a spring growth habit in 30 % accessions of this species [15, 21].

The host duplication of MITE in the first intron of *VRN-A1* resembles the TATA-box sequence (Fig. 3). A similar duplication is characteristic of a MITE inserted in the TATA-box of the dominant *VRN-A1a* allele of *T. aestivum* [13]. The last mutation may create an alternative promoter, or regulatory sites allowing modulation of transcription and, consequently, the vernalization requirement. Interestingly, the MITE insertion in *VRN-G1a* has a completely different duplication despite a high homology with the previous element (Fig. 3). Therefore, mobile elements are capable of inducing different modes of genetic regulation depending on their structure and region of insertion.

### Allelic variation in the *VRN-1* gene confirms the early divergence of *Ae. speltoides* from the progenitor of the B/G genomes

The two wild tetraploid wheat species, *T. dicoccoides* and *T. araraticum*, are relatively similar in plant morphology, but they differ in their genomic constitution (BBAA and GGAA, respectively). The identity of the donor(s) of the B and G genomes remains open to speculation. Based on the analysis of different nuclear and chloroplast loci, it was concluded that the ancestor of these genomes was a member of section *Sitopsis* of the genus *Aegilops* - most likely, *Ae. speltoides* Tausch [5, 7, 31]. Recently, we studied allelic variation within the *VRN-B1* gene of *T. dicoccoides* and found a low level of polymorphism compared with *VRN-A1* [15]. The similar conservatism was shown here for the *VRN-G1* locus of Timopheevi wheats, except the *VRN-G1a* allele was found to have no effect on vernalization requirements. The question then arose whether the structural peculiarities of the both loci were inherited from *Ae. speltoides,* the putative progenitor of B and G genomes.

Our results showed that divergence of the *VRN-1* locus in *Ae. speltoides* affected the annealing sites for primers usually used to target the corresponding *VRN-B1/G1* genes in polyploids. So, in the case of the promoter region, we used a newly designed pair of primers, while for the 1^st^ intron the D- genome specific pair was used (Table 1). In the both cases, PCR products were obtained corresponding to the recessive form of the *VRN-1* gene.

The sequencing of the *VRN-1* promoter region from 7 randomly selected accessions of *Ae. speltoides* showed almost no variation inside or near the probable regulatory sites, such as the TATA-box, VRN- and CArG-boxes etc., established earlier [10, 13, 18, 32, 33]. The majority of variation was found upstream of the regulatory sites, including a short tandem repeat AGCC track which consists of 2-3 repeat motifs in all the studied *Ae. speltoides* sequences, whereas in the known *VRN-A1*, *VRN-D1* and *VRN-B1/G1* sequences it includes 2, 3 and 4 repeats, respectively. Hence, there is a tendency toward an increasing number of repeats during evolution of the B/G- genome. Whether this tendency is due to some regulatory effect on *VRN-B1/G1* locus, or a result of stochastic processes, like genetic drift, is as yet unknown.

The *VRN-1* promoter sequences of *Ae. speltoides* were similarly homologous to the corresponding database sequences of wild tetraploids *T. dicoccoides* and *T. araraticum* with the maximum level of homology ~93 % in both cases. Of note, the latter sequences were almost identical (99-100 % homology) to those of hexaploid *T. aestivum*, implying a high conservation of *VRN-B1* promoter structure throughout both tetraploid and hexaploid stages of wheat evolution. In order to assess the relative rates of divergence between A-, B- and D- genomes of polyploids and corresponding diploid donors, we constructed a phylogenetic tree using the *VRN-1* promoter sequences analysed herein and those available in the public databases (Fig. 4). Divergence time was estimated using the molecular clock approach (see Methods). Based on this estimation, the progenitor of B/G genomes diverged from *Ae. speltoides* much earlier (~3.5 MYA), than the progenitor of A-genome did from *T. urartu*, closest to the diploid donor of A- genome, and slightly earlier than D- genome branch occurred (0.65 and 3.2 MYA, respectively). An intermediate position of the latter branch is consistent with the proposal of Marcussen et al. [2] that the ancestor of D- genome appeared due to a homoploid hybridization event between the progenitors of “A- and B- genome lineages”. The times of divergence of the main branches are earlier in our model as compared with the previous one, but close to the dates presented by Gill et al. [34] in the scheme of wheat phylogeny. Therefore, the obtained results confirm previous data indicating a fairly distant position of *Ae. speltoides* from the ancestors of polyploid forms and show that the *VRN-1* promoter region may serve as a good marker of phylogenetic relationships in the Triticeae tribe.

## Conclusions

In the present study we investigated variability in the promoter and 1^st^ intron regions of the vernalization gene *VRN-1* in different accessions of tetraploid species of Timopheevi group and their diploid G- genome progenitor, *Ae. speltoides*. Our results indicated that the occurrence of spring forms in tetraploid *T. timopheevii* is attributed to variation in the 1^st^ intron region of *VRN-A1* gene, namely, the insertion of a MITE element within “vernalization critical” parts of this region. All the studied accessions of *T. araraticum* with the winter growth type have no mutations of this kind despite the presence of other changes affecting regulatory regions in both *VRN-A1* and *VRN-G1* loci. This implies that the insertion of the MITE occurred in an ancestor of the modern *T. timopheevii* and has probably been selected during domestication of this species. The allelic variability of the *VRN-1* locus in the diploid *Ae. speltoides* was as low as in the tetraploid descendants to which the former species donated the B and G-genomes. The *VRN-1* promoter sequences were used to construct a phylogram which confirmed an early divergence of *Ae. speltoides* from the progenitor of the B/G genomes (3.6 MYA). Topology of the tree and the dates of divergence from the main branches are in agreement with other data, making the promoter region of *VRN-1* gene useful for targeting of different genomes and estimation of their phylogeny.

## Methods

### Plant material and DNA extraction

The plant material included tetraploid wheat species *T. araraticum* and *T. timopheevii* (45 and 4 accessions, respectively), and 23 accessions of *Ae. speltoides*. These accessions were selected from the different genebanks of Russia, Syria, Japan, and the USA (Additional file 1).

DNA was extracted from 7-day-old seedlings following [35]. Leaves from 3-5 seeds per accession were homogenised using a FastPrep-24 instrument (MP Biomedicals, USA).

### PCR

PCR primers reported in [13, 14, 23, 24] were used to identify different alleles of *VRN-A1* and *VRN-G1* loci in the material studied (Table 1; Fig. 1). To further discriminate different indels in the 1^st^ intron region of the *VRN-A1* locus we designed 2 pairs of primers: mitef/ miter and delf/ delr for detection of a 0.4 kb insertion and deletion of 2.7 kb, respectively. In the case of *Ae. speltoides* we designed the primers P8 and P10, instead of previously used P1/P5 primers, to amplify the *VRN-G1* promoter region covering ~900 bp upstream from the start codon (Table 1).

The PCR conditions were as described in [15]. Amplicons were separated through 1 % agarose gel.

### Sequencing of PCR products

Amplified DNA fragments were purified from an agarose gel using a QIAquick PCR purification kit (QIAGEN, Germany), then directly sequenced in both directions using an ABI PRISM Dye Terminator Cycle Sequencing ready reaction kit (Perkin Elmer Cetus, USA). Sequencing was conducted using resources of SB RAS Genomics Core Facilities (Novosibirsk, Russia, http://sequest.niboch.nsc.ru). GenBank accession numbers of *VRN-A1* and *VRN-G1* sequences from tetraploid species (KX344100-08 and KX344116-18, respectively) are partially represented in Table 2. The *VRN-1* promoter sequences of *Ae. speltoides* were deposited under Ac.№ KX344109-15.

### Sequence analysis

Multiple sequence alignments and the subsequent phylogenetic analysis were carried out using the ClustalW program and MEGA4 software [36, 37]. A phylogenetic tree was constructed using the Neighbor-Joining algorithm and 500 bootstrap replicates. Divergence time was assessed by MEGA4 using calibration against a previously established date of formation of the allotetraploid wheat of about 0.5-0.6 Million Years Ago (MYA) [1, 38].

### Evaluation of growth habit

Growth habits of the diploid and tetraploid accessions containing different *VRN-1* alleles were determined as described in [15] from two replicates in 2015-2016 (Table 2).

## Additional file

Additional file 1: Plant material used in the analysis, its origin and *VRN-1* genotype. (DOCX 26 kb)
