# Supplementary material for: The occurrence of spring forms in tetraploid Timopheevi wheat is associated with variation in the first intron of the VRN-A1 gene
Source: BMC Plant Biol. 2016 Nov 16;16(Suppl 3):107–18. doi: 10.1186/s12870-016-0925-y (PMC5123382; doi:10.1186/s12870-016-0925-y)
Supplement: Additional file 1: — Plant material used in the analysis, its origin and VRN-1 genotype. (DOCX 26 kb) [file 12870_2016_925_MOESM1_ESM.docx]

**Additional file 1**

**Plant material used in the analysis, its origin and *VRN-1* genotype**

| Accession no. | Origin | Growth habit* | Source** | | | *VRN-1* genotype*** |
| --- | --- | --- | --- | --- | --- | --- |
| ***T. araraticum*** | | | | | | |
| PI 427400 | Iraq | W | USDA-ARS | | | *VRN-A1f VRN-G1* |
| PI 427398 | Iraq | W | USDA-ARS | | | *VRN-A1f VRN-G1* |
| PI 538518 | Iraq | W | USDA-ARS | | | *VRN-A1f VRN-G1* |
| PI 427376 | Iraq | W | USDA-ARS | | | *VRN-A1f VRN-G1* |
| PI 427390 | Iraq | W | USDA-ARS | | | *VRN-A1f VRN-G1* |
| PI 427364 | Iraq | - | USDA-ARS | | | *VRN-A1f VRN-G1* |
| PI 427392 | Iraq | W | USDA-ARS | | | *VRN-A1f VRN-G1* |
| PI 427416 | Iraq | W | USDA-ARS | | | *VRN-A1f VRN-G1* |
| PI 427380 | Iraq | W | USDA-ARS | | | *VRN-A1f VRN-G1* |
| PI 427403 | Iraq | W | USDA-ARS | | | *VRN-A1f-del VRN-G1* |
| PI 538512 | Iraq | W | USDA-ARS | | | *VRN-A1f VRN-G1* |
| PI 427385 | Iraq | W | USDA-ARS | | | *VRN-A1f VRN-G1* |
| PI 427386 | Iraq | W | USDA-ARS | | | *VRN-A1f VRN-G1* |
| PI 538458 | Iraq | W | USDA-ARS | | | *VRN-A1f VRN-G1* |
| PI 538461 | Iraq | W | USDA-ARS | | | *VRN-A1f VRN-G1* |
| PI 427407 | Iraq | W | USDA-ARS | | | *VRN-A1f VRN-G1* |
| PI 427381 | Iraq | W | USDA-ARS | | | *VRN-A1f VRN-G1* |
| PI 538516 | Iraq | F | USDA-ARS | | | *VRN-A1f VRN-G1* |
| PI 654340 | Turkey | W | USDA-ARS | | | *VRN-A1f VRN-G1a* |
| Cltr 17680 | Iran | W | USDA-ARS | | | *VRN-A1f VRN-G1* |
| NSGC 861502 | Unknown | - | USDA-ARS | | | *VRN-A1f VRN-G1* |
| KU-8802 | Iraq | - | Kyoto University | | | *VRN-A1f VRN-G1* |
| KU-8739 | Iraq | - | Kyoto University | | | *VRN-A1f VRN-G1* |
| KU-8602 | Iraq | - | Kyoto University | | | *VRN-A1f VRN-G1* |
| KU-8913 | Turkey | - | Kyoto University | | | *VRN-A1f VRN-G1* |
| KU-8878 | Iraq | - | Kyoto University | | | *VRN-A1f VRN-G1* |
| KU-8822 | Iraq | - | Kyoto University | | | *VRN-A1f VRN-G1* |
| KU-8938 | Turkey | - | Kyoto University | | | *VRN-A1f VRN-G1* |
| KU-8926 | Turkey | W | Kyoto University | | | *VRN-A1f-del VRN-G1* |
| KU-1984B | Turkey | W | Kyoto University | | | *VRN-A1f VRN-G1a* |
| KU-8824A | Iraq | - | Kyoto University | | | *VRN-A1f VRN-G1* |
| KU-8944 | Iran | W | Kyoto University | | | *VRN-A1f-del VRN-G1* |
| KU-1964 | Turkey | W | Kyoto University | | | *VRN-A1f VRN-G1a* |
| K-28244 | Azerbaijan | W | VIR | | | *VRN-A1f VRN-G1* |
| K-31627 | Azerbaijan | W | VIR | | | *VRN-A1f VRN-G1* |
| TA 976 | Turkey | - | WGGR, KSU | | | *VRN-A1f VRN-G1* |
| TA 1008 | Turkey | W | WGGR, KSU | | | *VRN-A1f VRN-G1a* |
| TA 972 | Iraq | - | WGGR, KSU | | | *VRN-A1f VRN-G1* |
| IG 113296 | Iran | S | ICARDA | | | *VRN-A1f VRN-G1* |
| IG 116165 | Turkey | F | ICARDA | | | *VRN-A1f VRN-G1* |
| IG 116168 | Turkey | F | ICARDA | | | *VRN-A1f VRN-G1* |
| IG 116170 | Turkey | F | ICARDA | | | *VRN-A1f VRN-G1* |
| TRI 11509 | Iran | W | IPK | | | *VRN-A1f VRN-G1* |
| TRI 11507 | Iraq | W | IPK | | | *VRN-A1f VRN-G1* |
| TRI 17417 | Azerbaijan | W | IPK | | | *VRN-A1f-del VRN-G1* |
| ***T. timopheevii*** | | | | | | |
| K-29551 | Georgia | S | VIR | | *VRN-A1f-del/ins VRN-G1* | |
| K-29558 | Georgia | S | VIR | | *VRN-A1f-del/ins VRN-G1* | |
| PI 119442 | Turkey | S | USDA-ARS | | *VRN-A1f-del/ins VRN-G1a* | |
| ICG | Unknown,  provided by  E.B.Budashkina | S | Institute of  Cytology and Genetics  SB RAS | | *VRN-A1f-ins VRN-G1* | |
| ***Ae. speltoides*** | | | | | | |
| K-22 | Armenia | F | VIR | All accessions contain the recessive form of *VRN-1* according to PCR analysis of the main regulatory regions. | | |
| K-2278 | Iran | - | VIR |  |  |  |
| K-453 | Unknown | F | VIR |  |  |  |
| K-77 | Unknown | F | VIR |  |  |  |
| K-911 | Israel | - | VIR |  |  |  |
| K-1597 | Turkey | - | VIR |  |  |  |
| I-551352 | Syria | - | VIR |  |  |  |
| K-100 | Turkey | - | VIR |  |  |  |
| K-1018 | Unknown | - | VIR |  |  |  |
| K-66 | Unknown | F | VIR |  |  |  |
| K-452 | Unknown | F | VIR |  |  |  |
| K-3257 | Israel | - | VIR |  |  |  |
| K-1596 | Iran | - | VIR |  |  |  |
| I-570060 | Turkey | - | VIR |  |  |  |
| K-2371 | Unknown | - | VIR |  |  |  |
| K-2303 | Unknown | - | VIR |  |  |  |
| K-2276 | Turkey | - | VIR |  |  |  |
| K-443 | Unknown | - | VIR |  |  |  |
| K-1595 | Iran | - | VIR |  |  |  |
| K-48 | Unknown | F | VIR |  |  |  |
| K-1593 | Iran | - | VIR |  |  |  |
| K-1706 | Macedonia | - | VIR |  |  |  |
| K-1594 | Iran | - | VIR |  |  |  |

*- Growth habit is presented according to catalogues data, or evaluated in greenhouse (see Table 2). W- winter type; S- spring type; F- facultative, or intermediate between winter and spring types; “-“- no data;

** USDA-ARS- United States Department of Agriculture, Agricultural Research Service; WGGR, KSU- The Wheat GermPlasm Collection of Kansas State University, USA; VIR- N. I. Vavilov All-Union Research Institute of Plant Industry, St Petersburg, Russia; IPK- The Leibniz Institute of Plant Genetics and Crop Plant Research, Gatersleben, Germany; ICARDA- International Center for Agricultural Research in the Dry Areas.

*** *VRN-1* genotypes were established based on the PCR analysis and for some accessions (see Table 2) a partial sequencing of the promoter or 1^st^ intron regions was undertaken to confirm PCR results.
